# Dietary grape seed polyphenols repress neuron and glia activation in trigeminal ganglion and trigeminal nucleus caudalis

**DOI:** 10.1186/1744-8069-6-91

**Published:** 2010-12-10

**Authors:** Ryan J Cady, Jeffery J Hirst, Paul L Durham

**Affiliations:** 1 Center for Biomedical & Life Sciences, Missouri State University 524 N. Boonville, Springfield, MO, USA

## Abstract

**Background:**

Inflammation and pain associated with temporomandibular joint disorder, a chronic disease that affects 15% of the adult population, involves activation of trigeminal ganglion nerves and development of peripheral and central sensitization. Natural products represent an underutilized resource in the pursuit of safe and effective ways to treat chronic inflammatory diseases. The goal of this study was to investigate effects of grape seed extract on neurons and glia in trigeminal ganglia and trigeminal nucleus caudalis in response to persistent temporomandibular joint inflammation. Sprague Dawley rats were pretreated with 200 mg/kg/d MegaNatural-BP grape seed extract for 14 days prior to bilateral injections of complete Freund's adjuvant into the temporomandibular joint capsule.

**Results:**

In response to grape seed extract, basal expression of mitogen-activated protein kinase phosphatase 1 was elevated in neurons and glia in trigeminal ganglia and trigeminal nucleus caudalis, and expression of the glutamate aspartate transporter was increased in spinal glia. Rats on a normal diet injected with adjuvant exhibited greater basal levels of phosphorylated-p38 in trigeminal ganglia neurons and spinal neurons and microglia. Similarly, immunoreactive levels of OX-42 in microglia and glial fibrillary acidic protein in astrocytes were greatly increased in response to adjuvant. However, adjuvant-stimulated levels of phosphorylated-p38, OX-42, and glial fibrillary acidic protein were significantly repressed in extract treated animals. Furthermore, grape seed extract suppressed basal expression of the neuropeptide calcitonin gene-related peptide in spinal neurons.

**Conclusions:**

Results from our study provide evidence that grape seed extract may be beneficial as a natural therapeutic option for temporomandibular joint disorders by suppressing development of peripheral and central sensitization.

## Background

Temporomandibular joint disorder (TMJD) is a chronic disease that affects 15% of the adult population and is characterized by pain in the muscles and joint associated with mastication [1,2]. TMJD affects both men and women equally with the incidence of TMJD-related pain highest during adolescence age [3]. TMJD pathology involves activation of trigeminal ganglia nerves that provide sensory innervation to the muscles and joint and function to relay nociceptive signals to the trigeminal nucleus caudalis (TNC) [4,5]. In addition, development of peripheral and central sensitization, which are mediated by increased neuron-glia interactions, are implicated in TMJD pathology [6-8]. While peripheral sensitization of trigeminal nociceptive neurons involves satellite glial cells within the trigeminal ganglia, initiation and maintenance of central sensitization and persistent pain are facilitated by spinal astrocytes and microglia [9,10]. These glial cells modulate neuronal excitability by regulating the extracellular environment around neurons via the uptake of K^+ ^ions and glutamate by selective transporters [11,12].

Mitogen-activated protein (MAP) kinases, which are a family of signal transduction enzymes activated in response to inflammatory stimuli, play an important role in the development of peripheral and central sensitization [13-15]. The MAP kinase p38 is reported to mediate sensitization of primary and second order nociceptive neurons by increasing ion channel expression and activity, membrane receptor expression, and stimulating synthesis and secretion of cytokines that promote and maintain a hyperexcitable state of neurons [15-17]. Another protein implicated in the initiation of peripheral and central sensitization is the MAP kinase-responsive neuropeptide calcitonin gene-related peptide (CGRP) [18]. CGRP is known to stimulate glial cells in the trigeminal ganglia and TNC to release cytokines and other inflammatory molecules leading to a lower threshold of activation and contributing to persistent pain [19-21]. The stimulatory effects of CGRP are reported to involve upregulation of MAP kinases in neurons and glia [22-24]. In addition, CGRP promotes development of central sensitization by increasing the activity of glutamate receptors expressed on second order neurons that facilitate pain transmission [25,26].

The activity of MAP kinases is regulated by a family of proteins known as MAP kinase phosphatases (MKP) [27,28]. Expressions of MKPs, which are induced in response to inflammatory stimuli, function in a compensatory manner to restore normal levels of active MAP kinases. For example, MKP-1 is reported to preferentially suppress active, phosphorylated p38 levels in response to inflammatory stimuli or nerve injury [29]. Importantly, data from recent studies provide evidence that mice lacking the MKP-1 gene have elevated MAP kinase activity [30], increased cytokine-induced inflammation [29], and higher susceptibility to inflammatory injuries [31,32]. Thus, MKPs play an important role in modulating nociceptive responses to inflammatory stimuli and restoring cellular homeostasis [33].

Chronic pathological pain, which is characteristic of TMJD, is clinically difficult to treat using standard pharmacological therapies. However, natural products offer a novel method for preventing and treating chronic diseases [34,35]. In particular, results from a recent study provided evidence that including a polyphenol enriched grape seed extract (GSE) in the diet of mice significantly attenuated cognitive deterioration and Aβ oligomerization in neurons in a model of Alzheimer's disease [36]. The goal of our study was to investigate the effect of GSE as a dietary supplement on proteins in neurons and glia in trigeminal ganglia and TNC under basal conditions and in response to chronic inflammation of the temporomandibular joint (TMJ) induced by injection of complete Freund's adjuvant (CFA) in the joint capsule.

## Results

### Expression of MKP-1 in trigeminal ganglia is increased in response to GSE treatment

**Figure 1:**
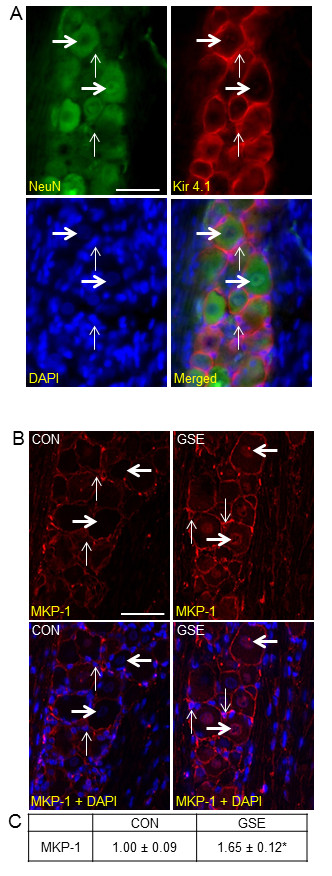
**Basal MPK-1 expression is increased in both neurons and satellite glia cells after GSE treatment**. (A) An image from a longitudinal section of trigeminal ganglia costained with the neuronal cell marker NeuN, the glial cell marker Kir4.1, or the nuclear dye DAPI is shown. The bottom right image shows a merged image of NeuN, Kir 4.1 and DAPI staining. Thick horizontal arrows identify the cell bodies of neurons while thin vertical arrows indicate satellite glia cells. (B) Sections of the posterolateral portion of the ganglion (V3) were obtained from untreated animals (CON) or animals treated with GSE for 14 days (GSE). Images of neuron-satellite glial cell enriched regions stained for MKP-1 are shown in the top panels. The bottom panels represent the same section costained for MKP-1 and DAPI. (C) The average fold change ± SEM of MKP-1 staining intensity from control values, which were made equal to one, is reported (n = 3 independent experiments) * *P *< 0.01 when compared to control. Magnification bar = 50 μm.

Initially, trigeminal ganglia from unstimulated control rats were isolated and longitudinal sections of the entire ganglia were co-stained with antibodies directed against NeuN and K_ir _4.1, and the fluorescent nuclear dye DAPI. NeuN protein staining was observed in the cytosol and nucleus of neuronal cell bodies in the trigeminal ganglion, while K_ir _4.1 staining was localized to the cytosol of satellite glial cells (Figure 1A). In trigeminal ganglia, numerous functional units consisting of a single neuronal cell body surrounded by satellite glia cells were visible throughout the mandibular (V3) region.

The cellular expression of the MAP kinase phosphatase MKP-1 in trigeminal ganglia obtained from animals drinking tap water (naïve control) or water supplemented with GSE was investigated by immunohistochemistry. In control animals, MKP-1 staining was primarily observed in the cytosol and nucleus of satellite glial cells, with staining barely visible in neurons (Figure 1B). However, the intensity of MKP-1 staining was greatly increased in the nucleus of neurons, and cytosol of satellite glial cells in animals treated with 200 mg/kg/d GSE. Animals treated with GSE had a significant increase in the staining intensity for MKP-1 (1.65 ± 0.12, *P *< 0.01) when compared to control animals (1.00 ± 0.09) (Figure 1C). Importantly, there were no appreciable changes in weight, eating/drinking habits, or grooming behavior in animals receiving GSE when compared to control animals (data not shown).

### GSE repressed CFA-stimulated P-p38 expression in trigeminal ganglia neurons

**Figure 2:**
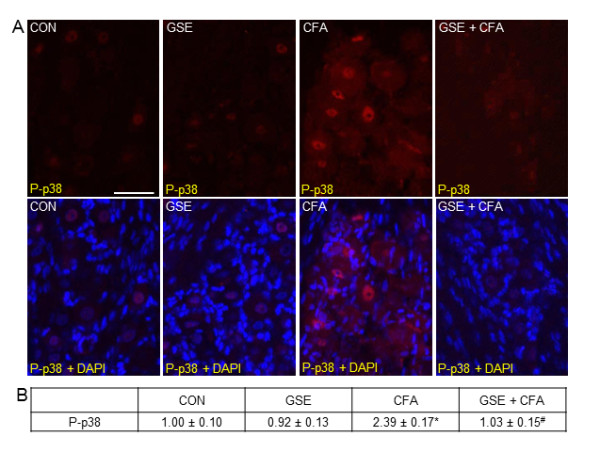
**Increased P-p38 expression in trigeminal ganglia neurons in response to CFA is repressed in rats on a GSE supplemented diet**. Sections of the posterolateral portion of the ganglion (V3) were obtained from untreated animals (CON), GSE treated animals (GSE), animals injected with CFA (CFA), or treated with GSE prior to injection of CFA (GSE + CFA). (A) Images of neuron-satellite glial regions stained for P-p38 are shown in the top panels. The bottom panels are the same sections co-stained for P-p38 and DAPI. (B) The average fold change ± SEM of P-p38 staining intensity from control values is reported (n = 3 independent experiments) * *P *< 0.01 when compared to control levels, while ^# ^*P *< 0.01 when compared to CFA stimulated levels. Magnification bar = 50 μm.

Expression of the active, phosphorylated form of p38 (P-p38) was barely visible in neurons or glia in ganglia obtained from control animals (Figure 2A). A similar staining pattern was observed in animals treated with GSE alone. In contrast, animals that received injections of 50 μL of CFA into their joint capsules had greatly increased nuclear and cytosolic P-p38 staining in neurons located in the V3 region of the ganglia seven days post injection. Importantly, a marked decrease in neuronal P-p38 expression to basal levels was seen in animals pretreated with GSE prior to injection of CFA. There was no noticeable difference in the staining intensity of ganglia obtained from animals treated with GSE alone (0.92 ± 0.13) when compared to control animals (1.00 ± 0.10). In contrast, a significant increase in staining intensity (2.39 ± 0.17, *P *< 0.01) was seen in response to CFA injection. However, rats pretreated with GSE prior to CFA injection had only a minor increase in staining intensity (1.03 ± 0.15) that was similar to control animals and significantly lower (*P *< 0.01) than animals with CFA injections (Figure 2B).

### GSE regulation of CGRP, MKP-1, and GLAST expression in the TNC

**Figure 3:**
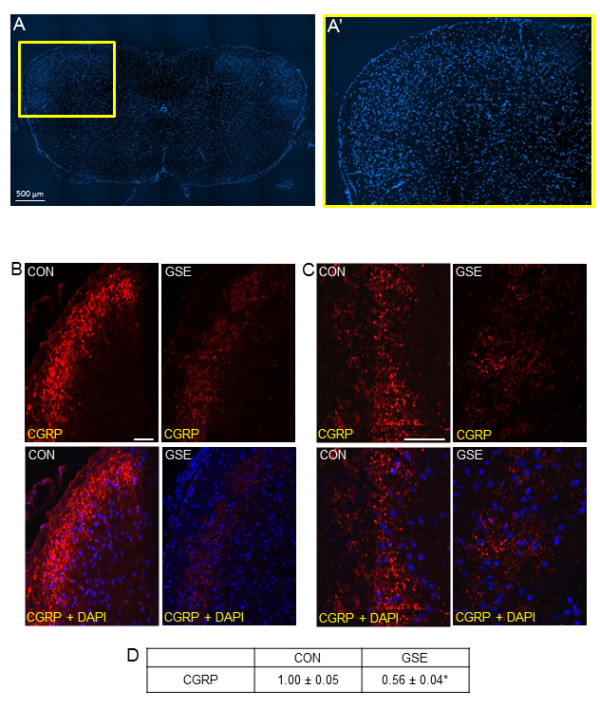
**GSE suppresses basal CGRP expression in the TNC**. (A) An image (40×) of a section of spinal cord 4 mm from the obex stained with the nuclear dye DAPI is shown. (A') An enlarged image of the area of spinal cord that contains the TNC (yellow box in A). (B) Sections of spinal cord obtained from untreated animals (CON) or treated with GSE for 14 days (GSE) and stained for CGRP are shown in the top panels (200×). The bottom panels represent a merged image of the same sections co-stained with CGRP and DAPI. (C) The top panels show images (400×) of TNC stained with antibodies against CGRP. The bottom panels represent the same sections stained for CGRP and DAPI. (D) The average fold change ± SEM of CGRP staining intensity from control values, which were made equal to one, is reported (n = 3 independent experiments) * *P *< 0.01 when compared to control. Magnification bar = 50 μm.

Initially, spinal cord from unstimulated animals was transversely sectioned 4-5 mm from the obex and stained with DAPI to identify the nuclei of neurons and glia in the TNC region (Figure 3A, A'). In unstimulated control animals, CGRP staining was readily detected in neurons located in discrete bands within the dorsal horn of the spinal cord, including the TNC region. However, the expression of CGRP in animals consuming GSE was greatly decreased when compared to control levels (Figures 3B and 3C). Inclusion of GSE in the water caused a significant decrease in CGRP staining intensity (0.56 ± 0.04, *P *< 0.01) when compared to control animals (1.00 ± 0.05) (Figure 3D).

**Figure 4:**
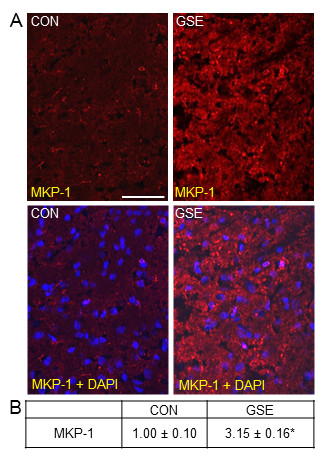
**GSE increases basal MKP-1 expression in neurons and glia in the TNC**. Sections of spinal cord were obtained from untreated animals (CON) or animals treated with GSE (GSE). (A) Images of a section of spinal cord stained for MKP-1 are shown in the top panels. The bottom panels represent the same sections co-stained with MKP-1 and DAPI. (B) The average fold change ± SEM of MKP-1 staining intensity from control values is reported (n = 3 independent experiments) * *P *< 0.01 when compared to control levels. Magnification bar = 50 μm.

The effect of GSE on basal MKP-1 expression in the TNC was similar to that observed in trigeminal ganglia. While the level of MKP-1 was barely detectable in neurons, astrocytes, and microglia in control animals, MKP-1 expression was greatly increased throughout the TNC in neurons and glia in response to GSE (Figure 4A). When compared to control animals (1.00 ± 0.10), GSE treated animals exhibited a significant increase in staining intensity (3.15 ± 0.16, *P *< 0.01) (Figure 4B).

**Figure 5:**
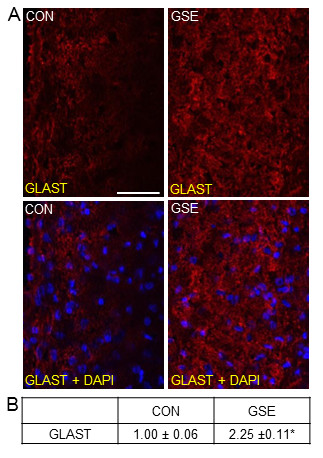
**Basal expression of GLAST is increased in glial cells in the TNC in response to GSE**. Sections of spinal cord were obtained from untreated animals (CON) and GSE treated animals (GSE). (A) Images of spinal cord sections stained for GLAST are shown in the top panels. The bottom panels represent the same sections co-stained with GLAST and DAPI. (B) The average fold change ± SEM of GLAST staining intensity from control values is reported (n = 3 independent experiments) * *P *< 0.01 when compared to control levels. Magnification bar = 50 μm.

A similar stimulatory effect was seen in the expression of the glial cell glutamate uptake transporter, GLAST, in the TNC of animals on a GSE supplemented diet. While GLAST staining was present in glia within the TNC in control animals, GLAST expression was greatly increased in glial cells throughout the TNC in animals treated with GSE (Figure 5A). When compared to control animals (1.00 ± 0.06), GSE treated animals exhibited a significant increase in staining intensity in glial cells (2.25 ± 0.11, *P *< 0.01) (Figure 5B).

### GSE repressed CFA-mediated expression of P-p38, GFAP and OX-42 in the TNC

**Figure 6:**
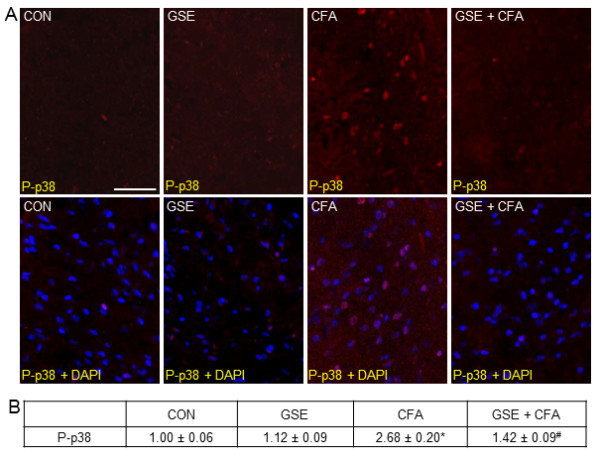
**GSE represses CFA stimulated P-p38 expression in TNC neurons and glial cells**. Sections of spinal cord were obtained from untreated animals (CON), animals treated with GSE (GSE), animals injected with CFA (CFA), or animals pretreated with GSE prior to an injection with CFA (GSE + CFA). (A) Images of sections of the TNC stained for P-p38 are shown in the top panels. The bottom panels represent the same sections co-stained P-p38 and DAPI. (B) The average fold change ± SEM of P-p38 staining intensity from control values is reported (n = 3 independent experiments) * *P *< 0.01 when compared to control levels while ^# ^*P *< 0.01 when compared to CFA stimulation. Magnification bar = 50 μm.

Minimal expression of P-p38 was detected in neurons and glia within the TNC obtained from control animals (Figure 6A). A similar staining pattern was seen in rats treated with GSE alone. In contrast, P-p38 expression was greatly increased in neurons and glia in the TNC in response to prolonged TMJ inflammation caused by injection of CFA into the joint capsule. However, levels of P-p38 were greatly repressed to near basal levels in animals receiving GSE supplemented in their water prior to CFA injection. To identify the cell types exhibiting increased P-p38 expression, tissues were costained with antibodies directed against the neuronal protein NeuN, the microglial protein Iba1, or an astrocyte marker glial fibrillary acidic protein (GFAP). Based on our costaining results, elevated levels of P-p38 were only observed in neurons and microglia (data not shown). There was no significant difference in the staining intensity in animals treated with GSE alone (1.12 ± 0.09) when compared to levels in control animals (1.00 ± 0.06) (Figure 6B). In contrast, CFA treatment caused a significant increase in P-p38 staining intensity (2.68 ± 0.20, *P *< 0.01) that was greatly repressed in animals receiving GSE (1.42 ± 0.09, *P *< 0.01).

**Figure 7:**
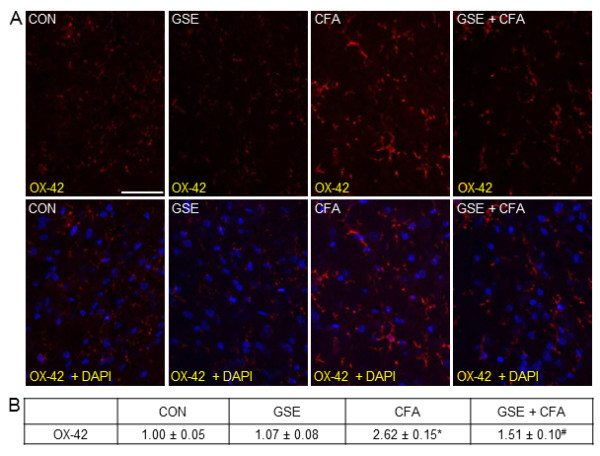
**GSE treatment represses CFA stimulated OX-42 expression in microglia**. Sections of spinal cord were obtained from untreated animals (CON), GSE treated animals (GSE), animals injected with CFA (CFA), or animals pretreated with GSE prior to an injection of CFA (GSE + CFA). (A) Images of spinal cord sections stained for OX-42 are shown in the top panels. The bottom panels represent the same sections co-stained with OX-42 and DAPI. (B) The average fold change ± SEM of OX-42 staining intensity from control values is reported (n = 3 independent experiments) * *P *< 0.01 when compared to control levels, while ^# ^*P *< 0.01 compared to CFA stimulation. Magnification bar = 50 μm.

The effect of CFA and GSE on microglia within the TNC was investigated using antibodies directed against OX-42, a marker of microglia activation. In tissues from control or GSE treated animals, low level expression of OX-42 was observed in microglia (Figure 7A). However, OX-42 expression was greatly increased in microglia in the TNC seven days after CFA injection into the joint capsule. In contrast, animals pretreated with GSE before CFA injection exhibited much lower levels of OX-42 expression when compared to animals that received CFA injections alone. There was no significant difference in the staining intensity of GSE treated animals (1.07 ± 0.08) when compared to levels in control animals (1.00 ± 0.05) (Figure 7B). In contrast, CFA injection resulted in a significant increase in staining intensity (2.62 ± 0.15, *P *< 0.01) that was repressed in animals pretreated with GSE prior to CFA injections (1.51 ± 0.10, *P *< 0.01).

**Figure 8:**
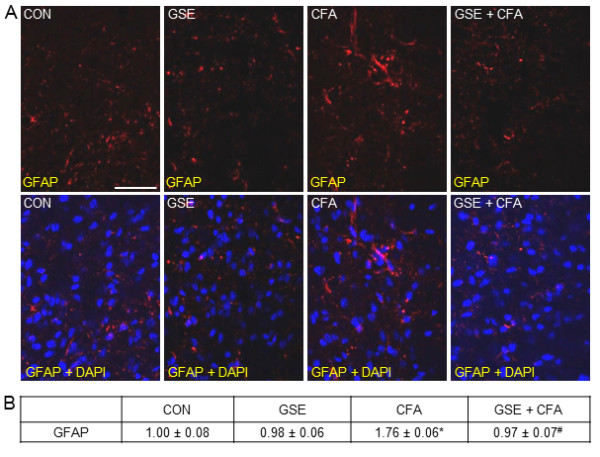
**GSE represses stimulated GFAP expression in astrocytes in response to CFA**. Sections of spinal cord were obtained from untreated animals (CON), GSE treated animals (GSE), animals injected with CFA (CFA), or animals pretreated with GSE prior to an injection of CFA (GSE + CFA). (A) Images of spinal cord sections stained for GFAP are shown in the top panels. The bottom panels represent the same sections co-stained with GFAP and DAPI. (B) The average fold change ± SEM of GFAP staining intensity from control values is reported (n = 3 independent experiments) * *P *< 0.01 when compared to control levels, while ^# ^*P *< 0.01 compared to CFA stimulation. Magnification bar = 50 μm.

Low level expression of GFAP, a marker of activated astrocytes, was observed in numerous astrocytes in the TNC from control animals and those treated with GSE (Figure 8A). In contrast, GFAP expression was greatly increased in response to CFA injections. However, the stimulatory effect of CFA on GFAP expression was greatly repressed in animals receiving with GSE prior to CFA. There was no significant difference in the staining intensity of rats treated with GSE (0.98 ± 0.06) when compared to control animals (1.00 ± 0.08) (Figure 8B). In contrast, CFA caused a significant increase in staining intensity (1.76 ± 0.06, *P *< 0.01) over levels in control and GSE treated animals. The stimulatory effect of CFA on GFAP levels was significantly repressed in animals on a GSE supplemented diet (0.97 ± 0.07, *P *< 0.01).

## Discussion

In this study, we initially investigated cellular changes in trigeminal neurons and glia in response to GSE supplemented diets. We chose to study the effect of MegaNatural-BP GSE, a highly purified extract from *Vitis vinifera *seeds containing >90% polyphenols as gallic acid equivalents [36]. Inclusion of this product in the diet of mice was reported to significantly attenuate cognitive deterioration and Aβ oligomerization. Importantly, the concentration of GSE shown to cause changes in Aβ oligomerization was identical to the amount used in our study and shown to suppress inflammatory responses caused by prolonged stimulation of trigeminal nerves. GSE supplemented diets significantly increased the basal expression of MKP-1 in both neurons and glia within the trigeminal ganglia, as well as in neurons and glia in the TNC. MKP-1 is reported to dephosphorylate, and therefore, inactivate several MAP kinases including p38, JNK, and ERK, in a concentration dependent manner [33]. Thus, increased expression of MKP-1 in trigeminal ganglia and the TNC would function to control MAP kinase activity in response to trigeminal nerve activation. In support of this notion, we found that CFA stimulated levels of active p38 in trigeminal ganglia and TNC were significantly repressed in animals receiving GSE when compared to control animals. Importantly, MAP kinases such as p38, are implicated in peripheral and central sensitization, and blocking MAP kinases with specific inhibitors is reported to suppress nociceptive responses and sensitization [15,37,38]. To our knowledge, this is the first study to provide evidence that GSE can lead to sustained, elevated levels of MKP-1, an enzyme that plays a fundamental role in regulating the activity of MAP kinases and thus, controlling development of peripheral and central sensitization.

Our finding that natural products such as GSE increases MKP-1 expression is in agreement with our previous result that demonstrated animals fed an enriched diet containing 1% or 10% cocoa exhibited elevated levels of MKP-1 [39]. Interestingly, the anti-inflammatory effects of dexamethasone have been shown to be meditated, at least to some degree, by inducing a sustained expression of MKP-1 [40]. Furthermore, the anti-nociceptive effects of opioids are reported to involve increased expression of MKP-1 [41]. Thus, it appears that daily consumption of GSE or cocoa may function to block inflammatory and nociceptive responses in the trigeminal ganglion by a similar cellular mechanism as has been reported for glucocorticoids and opioids.

Another interesting finding from our study was that dietary GSE decreases basal expression of the MAP kinase-responsive gene CGRP, while increasing expression of GLAST in the TNC. CGRP is a multifunctional neuropeptide known to promote inflammatory and nociceptive responses and is implicated in TMJ pathology [18]. In response to trigeminal neuron activation, peripheral CGRP release promotes an inflammatory response within the joint capsule, while central release of CGRP mediates enhanced nociception and central sensitization. CGRP is thought to contribute to nociception and central sensitization by its stimulatory effects on second order neurons and glia within the TNC [42,43]. For example, CGRP release causes upregulation of α-amino-3-hydroxyl-5-methyl-4-isoxazole-propionate (AMPA) receptor proteins [44], prolongs activity of substance P by preventing its degradation [45], and increases levels of the purinegic receptor P2X_3 _[46]. In addition, CGRP via activation of protein kinase A (PKA) is proposed to modulate activity of (N-methyl-D-aspartic acid) NMDA receptors thereby regulating glutamate excitation of second order nociceptive neurons [25]. Thus, decreased levels of CGRP are likely to reduce the stimulatory effects of glutamate, substance P, and ATP on second order neurons that promote nociception [25,47]. Significantly, we also found that GSE caused increased expression of the glutamate transporter GLAST that functions to regulate the amount of extracellular glutamate. Sustained GLAST expression in astrocytes leads to reduced glutamate levels, thus decreasing the excitability state of neurons and minimizing spontaneous activation [48]. To our knowledge, this is the first example of using natural products as dietary supplements to increase GLAST expression. Taken together, our findings provide evidence that GSE functions to regulate levels of proteins known to play important roles in regulating spinal neuronal and glial excitability by decreasing CGRP expression and increasing GLAST expression.

In our study, we observed increased expression of P-p38 in trigeminal ganglia neurons, and neurons and microglia in the TNC in response to CFA injection. Increased levels of P-p38 are thought to contribute to the development of peripheral and central sensitization [10,16], a process characteristic of TMJ pathology, by increasing the synthesis and release of proinflammatory cytokines [49,50]. Chronic stimulation by CFA is a well-established model for TMJ pathology and when injected into the joint capsule results in a long-lasting inflammatory event and prolonged activation of nociceptive neurons [9,51,52]. Importantly, we found that the stimulatory effects of CFA on P-p38 expression in trigeminal ganglia and TNC were significantly repressed in animals receiving GSE. We propose that the basal increase in MKP-1 mediated by dietary GSE is responsible, at least in part, for the repressed expression of P-p38 in trigeminal ganglia and TNC neurons and glia in response to CFA since MKP-1 is a primary regulator of p38 activity [33]. Taken together, GSE repression of P-p38 is likely to prevent development of a persistent pain state in response to CFA, which is used as a model of TMJ pathology.

We found that bilateral injection of CFA caused enhanced activity of astrocytes and microglia and that pretreatment with GSE was sufficient to repress the stimulatory effect of CFA on these glial cells. Our results with respect to CFA are in agreement with previous studies that reported the inflammatory response to CFA injection increased activation of astrocytes and microglia, as demonstrated by increases in the expression of GFAP and OX-42, respectively [5,9,53]. While once thought to play mainly a supportive role for neurons, it is now known that astrocytes and microglia modulate the activation state of neurons and contribute to hypersensitivity and the chronification of pain states [54]. Under normal conditions, both microglia and astrocytes are relatively quiescent. However, in response to noxious/inflammatory stimuli these cells release proinflammatory cytokines leading to activation of nearby neurons and other glia [55,56]. Interestingly, GSE is able to repress the stimulatory effects of CFA in both astrocytes and microglia as demonstrated by reduced levels of GFAP, a structural protein and a marker of astrocyte activation, and OX-42, a marker of activated microglia [57,58]. Enhanced cellular activity of both astrocytes and microglia in the trigeminal system have been implicated in the development of persistent pain states [59]. Thus, it appears that GSE functions to modulate cellular responses to inflammatory stimuli by inhibiting glial cell activation, which in turn, would decrease the excitability state of nociceptive neurons in the TNC.

## Conclusions

In summary, GSE elevated expression of proteins in trigeminal ganglia and TNC known to decrease neuronal excitability and suppressed CFA-induced expression of several proteins implicated in peripheral and central sensitization. Although not investigated in our study, inclusion of dietary GSE would likely also suppress CFA-mediated nocifensive behaviors, including mechanical allodynia and thermal hyperalgesia, which are characteristic of the development of central sensitization. Thus, based on our cellular findings and results from behavioral studies on CFA-induced inflammation in the TMJ or masseter muscle [60,61], we propose that dietary GSE will be therapeutically beneficial for reducing chronic inflammatory pain associated with TMJ disorders.

## Methods

### Animals

All animal studies were approved by the Institutional Animal Care and Use Committee at Missouri State University and were conducted in compliance with all established guidelines in the Animal Welfare Act and National Institutes of Health. A concerted effort was made to reduce the number of animals used and to minimize any suffering. Adult male Sprague-Dawley rats (200-230 g), Charles River Laboratories, Wilmington, MA) had unrestricted access to food and water and were kept on a 12-hour light/dark cycle. Changes in food and water consumption as well as weight and grooming behaviors were monitored and recorded daily to assess the overall health of the animals. Rats were placed into two groups with one group of rats receiving a normal diet while the other group received a normal diet supplemented with 200 mg/kg/d MegaNatural-BP Grape Seed Polyphenol Extract (GSE, Healthy Origins, Pittsburgh, PA) dissolved in their drinking water. This amount is equivalent to a human dose of 1 g/d using the Food and Drug Administration criteria for converting drug equivalent dosages across species [36]. Drinking water for all animals was changed every two days.

### Chronic model of TMJ inflammation

Rats were anesthetized by inhalation of 3.5% isoflurane (VetEquip, Pleasanton, CA). After fourteen days of drinking normal water or GSE supplemented water, some rats received an injection of 50 μL complete Freund's adjuvant (CFA; Sigma-Aldrich, St. Louis, MO) in each TMJ capsule. All animals then continued their assigned diets for seven more days before being sacrificed. The time of treatment, preparation, and amount of stimulatory agent were based on results from previous studies in our laboratory [39,62]. As controls, some animals were not injected (naïve control).

### Tissue isolation and preparation

Trigeminal ganglia and spinal cord encompassing the spinomedullary junction (Vc/C1) transition zone containing the TNC, were removed from all rats following CO_2 _asphyxiation and placed in a solution of 4% paraformaldehyde overnight. Following paraformaldehyde fixation, tissues were incubated in 15% sucrose in distilled water at 4°C for 1 hour and then 30% sucrose overnight at 4°C. Trigeminal ganglia and spinal cord tissues were then placed dorsal side up on a slide, covered with OCT Compound (Sakura Finetek, Torrance, CA) and quickly frozen. Fourteen-micron longitudinal sections of the entire trigeminal ganglion tissue were serially prepared using a cryostat (Microm HM 525, Thermo Scientific, Waltham, MA) set at -20°C. Spinal cord sections (20 μm) containing the TNC were sectioned transversely at a distance of 4-5 mm posterior to the obex using a cryostat set at -18°C. All sections were mounted on Superfrost Plus microscope slides (Fisher Scientific, Pittsburg, PA). Each slide used for immunohistochemistry contained one section from each experimental condition.

### Immunohistochemistry

Slides containing either sectioned ganglia or spinal cord were incubated in a solution of 0.1% Triton X-100 and 5% donkey serum (Jackson Immuno Reasearch Laboratories, West Grove, PA) for 30 minutes. Next, sections were incubated overnight at 4°C with rabbit polyclonal antibodies directed against the phosphorylated, active MAP kinase protein P-p38 (1:200 in 5% donkey serum/PBS, Cell Signaling, Beverly, MA), MKP-1 (1:500, Upstate Cell Signaling Solutions, Lake Placid, NY), CGRP (1:1000, Sigma-Aldrich), K_ir _4.1 (1:1000, Alomone Labs, Jerusalem, Israel), or GLAST (1:200, Abcam, Cambridge, MA). In addition, some sections were incubated for three hours at room temperature with either mouse monoclonal antibodies against NeuN (1:500, Millipore, Billerica, MA), OX-42 (1:200, Abcam), or GFAP (1:500, Millipore), or a goat polyclonal antibody against Iba1 (1:300, Abcam). Sections were next incubated for one hour at room temperature with either Alexa Fluor 594 donkey anti-rabbit (P-p38, MKP-1, CGRP, K_ir _4.1, GLAST), Alexa Fluor 594 donkey anti-mouse (OX-42, GFAP), Alexa Fluor 488 donkey anti-mouse (NeuN), or Alexa Fluor 488 donkey anti-goat (Iba1) IgG antibodies (diluted 1:500 in PBS, Invitrogen, Carlsbad, CA) to detect immunofluorescent proteins by UV-fluorescence microscopy. Sections were mounted using Vectashield medium (H-1200) containing 4',6-diamidino-2-phenylindole (DAPI; Vector Laboratories, Burlingame, CA) to co-stain cell nuclei. Images (40×, 200×, and 400×) were collected using a Zeiss Axiocam mRm camera mounted on a Zeiss Imager Z1 fluorescent microscope equipped with an ApoTome. Image acquisition was performed using Zeiss AxioVision (Rel 4.7, Thornwood, NY).

### Measurement of cell counts and staining intensity

Images containing the mandibular branch (V3) of the middle portion of the trigeminal ganglion or regions of spinal cord tissue containing the TNC were used for analysis. Four randomly chosen 400× images containing a similar number of cells as identified by DAPI, were analyzed for each experimental condition, which were repeated in three independent experiments, resulting in 12 images for the intensity measurements. The relative staining intensity measurements were based on our previously published protocols [39,63]. Two researchers blinded to the experimental conditions performed the measurements. For images from trigeminal ganglia, the mean gray intensity of four circular regions from areas containing a single neuron and associated surrounding satellite glial cell were measured and the mean gray intensity from an area containing only Schwann cells and fiber tracts was subtracted as background. The staining intensity in spinal cord tissue was determined by measuring the mean gray intensity from four regions of staining in the trigeminal nucleus caudalis and subtracting the intensity from areas containing only background staining. The fold change in staining intensity was defined as the mean change in relative intensity in the experimental condition when compared to mean levels of the unstimulated control tissue, which was set equal to one. Statistical analysis was performed using either one way ANOVA followed by post-hoc Tukey test or Students T-test. Results were considered significant when *P *< 0.05. All statistical tests were performed using SPSS (Version 16, IBM, Chicago, IL).

## Competing interests

The authors declare that they have no competing interests.

## Authors' contributions

RC performed the experiments and data analysis, and helped draft the manuscript. JH helped with experiments and data analysis. PD conceived of the study, participated in its design and coordination, and helped to draft and edit the manuscript. All authors read and approved the final manuscript.
